# Time-dependent vaccine efficacy estimation quantified by a mathematical model

**DOI:** 10.1371/journal.pone.0285466

**Published:** 2023-05-11

**Authors:** Jennifer Loria, Vinicius V. L. Albani, Francisco A. B. Coutinho, Dimas T. Covas, Claudio J. Struchiner, Jorge P. Zubelli, Eduardo Massad

**Affiliations:** 1 Instituto de Matemática Pura e Aplicada, Rio de Janeiro, Brazil; 2 School of Mathematics, Universidad de Costa Rica, San José, Costa Rica; 3 LAMMCA, Department of Mathematics, Federal University of Santa Catarina, Florianopolis, Brazil; 4 Department of Pathology, University of São Paulo, São Paulo, Brazil; 5 Instituto Butantan, São Paulo, Brazil; 6 School of Applied Mathematics, Fundação Getúlio Vargas, Rio de Janeiro, Brazil; 7 Mathematics Department, Khalifa University, Abu Dhabi, UAE; 8 School of Medicine, University of São Paulo and LIM01-HCFMUSP, São Paulo, Brazil; Khalifa University of Science Technology - Abu Dhabi Campus: Khalifa University of Science and Technology, UNITED ARAB EMIRATES

## Abstract

In this paper we calculate the variation of the estimated vaccine efficacy (VE) due to the time-dependent force of infection resulting from the difference between the moment the Clinical Trial (CT) begins and the peak in the outbreak intensity. Using a simple mathematical model we tested the hypothesis that the time difference between the moment the CT begins and the peak in the outbreak intensity determines substantially different values for VE. We exemplify the method with the case of the VE efficacy estimation for one of the vaccines against the new coronavirus SARS-CoV-2.

## Introduction

Vaccines play a central role in our aptitude to prevent diseases [1]. Indeed, in a highly connected world, it acts as a fundamental tool to decrease the multiscale interaction that enhances the propagation of viruses between individuals and populations [2, 3]. For the last couple of centuries, vaccine campaigns have proved to be the most effective medical strategy to reduce death and morbidity caused by infectious diseases [4]. It has been estimated that, before the advent of COVID-19, vaccines saved at least 2–3 million lives per year worldwide [5]. Vaccines differ from classical medicinal products, because the intention of a vaccine is to prevent an infection and/or a disease in a healthy population [1]. Therefore, the benefit of the vaccine ought to significantly outweigh any risks. To establish both safety and efficacy Clinical Trials (CT) must then be undertaken [6]. CTs are divided in three major phases: Phase 1 clinical trials are to test the initial safety and tolerability of the candidate vaccine; Phase 2 clinical trials test the safety, immunogenicity, and dose ranges; and Phase 3 clinical trials are for additional safety data, immunogenicity, and efficacy [7]. The most important phase of the trial is the third phase since it is required by regulatory agencies to aprove the vacinne for public use. [13].

The decision made by regulatory agencies on whether to license or not a vaccine is made based on CT results combined with data on the vaccine manufacture. The regulatory agency also provides, based on such data, the vaccine usage information, such as indications, contraindications, and precautions. Once a vaccine is licensed, recommendations on its use are released by the immunization advisory bodies that must be followed in the program implementation. This ensures that those who are recommended to be vaccinated actually receive the vaccine. Furthermore, modifications in the vaccine use policy can be implemented based on post-licensing surveillance data on vaccine safety and effectiveness [6, 8–12]. The objective of this work is to simulate different scenarios for the third phase of clinical trials. To meet this objective, in this paper, the third phase of clinical trials is organized as follows:

1. A group of volunteers who never had the disease is randomly selected from the population. We call this number *S*(0).
2. This group is divided into two subgroups, a fraction *qS*(0) who will be injected with the vaccine and a fraction (1 − *q*)*S*(0) who will be injected with placebo. We exclude those individuals that get the infection before being vaccinated.
3. The trial begins at times *t*_*c*_ and *t*_*p*_, when vaccine and placebo, respectively, begins to be injected. Ideally *t*_*c*_ = *t*_*p*_, but, for logistic reasons, this may not be so. The beginning of the trial is *t*_*c*_ or *t*_*p*_, depending on the one that occurs first.

If vaccine efficacy (*VE*) could be evaluated as 1 minus some measure of relative risk (*RR*) in the vaccinated group compared with the unvaccinated group [13, 14].

We shall elaborate later the biological significance on the vaccine efficacy and possible alternatives in the One Possible Way to Explain Vaccine Efficacy Subsection.

VE can vary substantially in different settings [15], being influenced by many factors, including the time variation in the force of infection (also known as incidence density rate), defined as the per capita rate at which susceptible individuals in a population acquire the infection per unit of time [16, 17]. Hence, in endemic situation, the force of infection does not vary significantly in time and the calculation of *VE* is straightforward and constant in time. When the infection is new or recurrent, or present an important seasonal pattern, however, the moment along the outbreak course may result in different values of *VE*.

The purpose of this paper is to calculate the variation of the estimated *VE* due to the time difference between the moment the CT begins and the peak in the outbreak intensity. In the simple model presented in A Deterministic Model Subsection, substantial differences occur in the estimation in the values for *VE*, which are exemplified with one of the vaccines against the new virus SARS-CoV-2.

## Materials and methods

### One possible way to explain vaccine efficacy

In this section we propose a simple model to explain what vaccine efficacy is. This model considers that a test is performed ending with INFECTED-VACCINATED individuals and INFECTED-NOT-VACCINATED individuals.

Let λ(*t*) be the *per capita* incidence in this outbreak and *S*(0) be the number of individuals that are involved at the beginning of this trial.

We now define the vaccination efficacy (VE) and connect it to the relative risk (RR) that will play a crucial role throughout this paper:

$$\begin{array}{c} VE=1-RR. \end{array}$$

In this paper, *RR* is defined as

$$\begin{array}{c} RR=\frac{ARV}{ARU}, \end{array}$$

where, *ARV* and *ARU* are defined as follows, let *a* be the number of cases among persons who received the vaccine and *b* the number of non-cases among vaccinated persons. Let *c* be number of cases among people injected with placebos and *d* the number of non-cases among this group. Then

$$\begin{array}{c} ARV=\frac{a}{a+b}\;\;\text{and}\;\;ARU=\frac{c}{c+d}. \end{array}$$

The parameters described above can be better appreciated in Table 1:

**Table 1:** Parameters used in the evaluation of the vaccine efficacy.

	Cases	Non-Cases	Total	Risk
Vaccinated	*a*	*b*	*a*+ *b*	$ARV=\frac{a}{a+b}$
Unvaccinated	*c*	*d*	*c*+ *d*	$ARU=\frac{c}{c+d}$

From the inputs of Table 1, the vaccine efficacy is estimated as follows [13, 14]:

$$\begin{array}{c} VE=1-RR=1-\frac{ARV}{ARU}, \end{array}$$ (1)

where *ARV* is the fraction of persons in the trial injected with the vaccine that got the infection and *ARU* is the fraction of the persons injected with placebo that got the infection. Of course, if *ARV* = *ARU*, the *VE* is zero and the vaccine is useless. On the other hand, if *a* in the definition of *ARV* is zero, then *VE* = 1, and the vaccine is perfect. However, as illustrated by the results below, these two extreme cases are difficult to happen in practice. Let us first examine in more detail the significance of ARV and ARU with a simple calculation. Assume that a, b, c, and d are real variables and not integers. This is a common practice in mathematical biology and will be relaxed below. This type of model is called deterministic. Deterministic models can also be used, for example, to predict disease incidence, to estimate the underreport of infections, and access the impact in the delay of vaccination strategies [18–22].

Assume that we have selected for the trial *N*_*v*_ vaccinnated persons and *N*_*p*_ persons to inject with placebo. Then, clearly *a*+ *b* = *N*_*v*_ at any moment since we are assuming no deaths. Also, *c*+ *d* = *N*_*p*_. Of course, during the trial *a*, *b*, *c*, and *d* vary with time. Assume that the force of infection for vaccinated individuals is λ_*v*_. Then, *a*(*t*) obeys

$$\begin{array}{c} \frac{da}{dt}=\lambda_{v}\left(N_{v}-a\right), \end{array}$$ (2)

whose solution is

$$\begin{array}{c} a\left(t\right)=N_{v}\left(1-{\text{e}}^{-\lambda_{v}t}\right). \end{array}$$ (3)

Then,

$$\begin{array}{c} ARV\left(t\right)=1-{\text{e}}^{-\lambda_{v}t}. \end{array}$$ (4)

Similarly, assuming that the force of infection among the placebo population is λ_*p*_ gives

$$\begin{array}{c} ARU\left(t\right)=1-{\text{e}}^{-\lambda_{p}t}. \end{array}$$ (5)

If λ_*v*_ < λ_*p*_, we have the ratio

$$\begin{array}{c} \frac{ARV(t)}{ARU(t)}=\frac{1-{\text{e}}^{-\lambda_{v}t}}{1-{\text{e}}^{-\lambda_{p}t}}, \end{array}$$ (6)

where *t* is the time the trial ends because of a certain criterion and *ARV*(*t*)/*ARU*(*t*) is a number between 0 and 1. When λ_*v*_ is zero, *ARV*(*t*)/*ARU*(*t*) = 0, which means that the vaccine is perfect and *VE* = 1, but if λ_*v*_ = λ_*p*_, *VE* = 0 and the vaccine is useless.

Note that if λ_*v*_ > λ_*p*_, we have a vaccine efficacy negative, which means that the vaccine is producing the disease!

In the next section we present a more elaborate model to show how the results of the Phase 3 of the clinical trial depend on the outbreak intensity and the moment in the outbreak when the test is conducted. The model also allows one to compare the result of the test with a pre-determined nominal (true) vaccine efficacy.

### A deterministic model

Since a stochastic version of the model as described above is very cumbersome, we shall present only a deterministic version.

The model is an extension of the classical SI models without demography [23] and uses the following variables:

1. *Susceptible* (*S*(*t*)): Number of individuals which can either be vaccinated or receive a placebo shot or they may even acquire the infection before receiving the vaccine or placebo.
2. *Vaccinated* (*V*(*t*)): Number of individuals who are transferred from the susceptible state and administered the vaccine.
3. *Failure* (*F*(*t*)): Number of individuals who represent the fraction of susceptible vaccinates who fail to be immunized (that is, not completely protected after vaccination) and can acquire infection with the same rate λ(*t*) as those non-vaccinated susceptible.
4. *Protected* (*P*(*t*)): Number of individuals who received the vaccine and got protected against the infection.
5. *Placebo* (*Pl*(*t*)): Number of individuals who received the placebo shot and are subject to the same risk of infection as the susceptible individuals.
6. *Infective* (*I*(*t*)): Number of individuals infected before receiving either the vaccine or the placebo.
7. *Infected placebo* (*I*_*p*_(*t*)): Number of individuals who received the placebo and got the infection with the same rate λ(*t*) as the other infected individuals.
8. *Infected vaccinated* (*I*_*v*_(*t*)): Number of individuals who received the vaccine but were not immunized and got the infection with the same rate λ(*t*) as the other infected.

**Fig 1:**
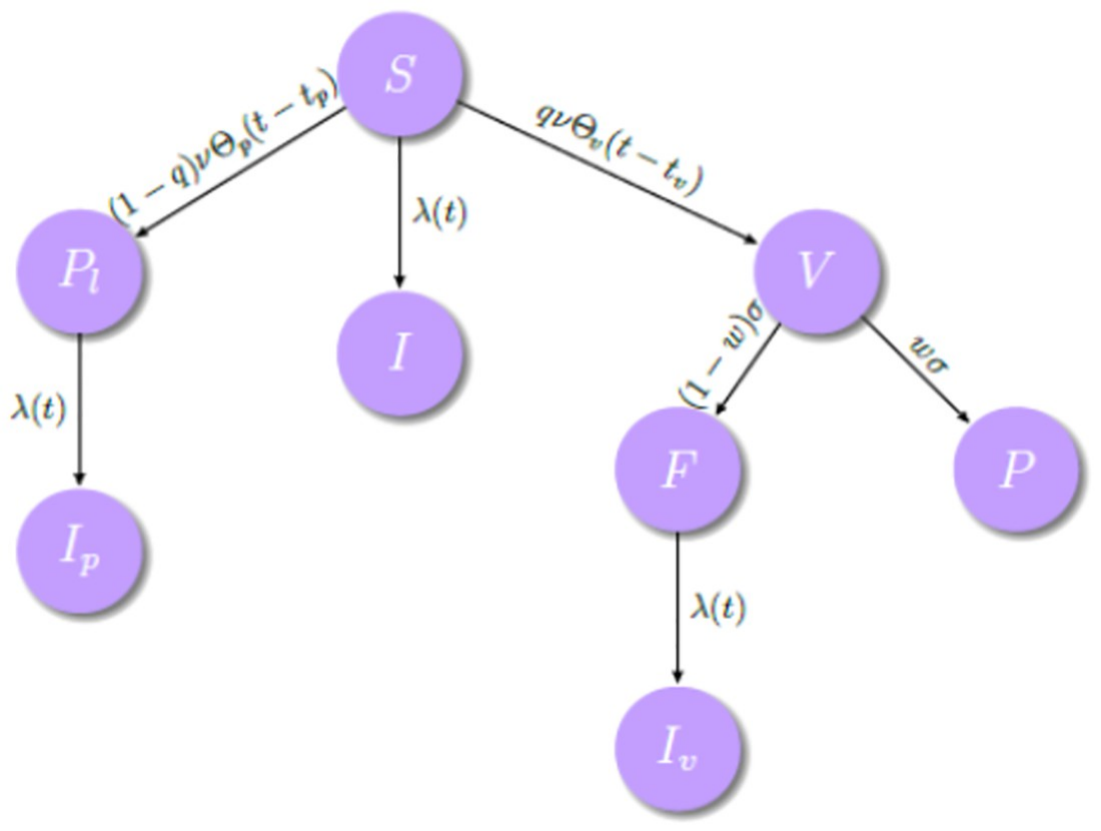
Schematic representation of the epidemiological model representing its different compartments.

Fig 1 shows a diagram with the model’s compartments and transitions.

Notice that the three infected compartments (*I*, *I*_*p*_ and *I*_*v*_) are independent of each other. In the case of compartment I, we have infected individuals before receiving placebo or vaccine. Thus, they are not able to participate in the clinical trial and for this reason this compartment is not part of most of the analyses.

It is worth noticing that the model does not consider demography because the cohort is fixed in size and because the duration of the clinical trial is too short relative to the life-span of its components.

The model is described by the following equations:

$$\begin{array}{c} \frac{dS}{dt}\left(t\right)=-S\left(t\right)\left(q\nu \Theta_{v}\left(t-t_{v}\right)+\left(1-q\right)\nu \Theta_{p}\left(t-t_{p}\right)+\lambda \left(t\right)\right), \end{array}$$ (7)

$$\begin{array}{c} \frac{dI}{dt}\left(t\right)=\lambda \left(t\right)S\left(t\right), \end{array}$$ (8)

$$\begin{array}{c} \frac{dV}{dt}\left(t\right)=S\left(t\right)q\nu \Theta_{v}\left(t-t_{v}\right)-\sigma V\left(t\right), \end{array}$$ (9)

$$\begin{array}{c} \frac{dPl}{dt}\left(t\right)=S\left(t\right)\left(1-q\right)\nu \Theta_{p}\left(t-t_{p}\right)-\lambda \left(t\right)Pl\left(t\right), \end{array}$$ (10)

$$\begin{array}{c} \frac{dI_{p}}{dt}\left(t\right)=\lambda \left(t\right)Pl\left(t\right), \end{array}$$ (11)

$$\begin{array}{c} \frac{dF}{dt}\left(t\right)=-\lambda \left(t\right)F\left(t\right)+\left(1-w\right)\sigma V\left(t\right), \end{array}$$ (12)

$$\begin{array}{c} \frac{dI_{v}}{dt}\left(t\right)=\lambda \left(t\right)F\left(t\right), \end{array}$$ (13)

$$\begin{array}{c} \frac{dP}{dt}\left(t\right)=w\sigma V\left(t\right), \end{array}$$ (14)

where λ is the force-of-infection (described below), *q* is the daily proportion of susceptible individuals who receive the vaccine, *ν* is the daily administration rate, either of vaccine or placebo, *Θ*_*v*_(*t* − *t*_*v*_) and *Θ*_*p*_(*t* − *t*_*p*_) are Heaviside functions that determines when the vaccine and the placebo administration start, *σ* is the rate with which vaccinated individuals get fully protected and *ω* is the true efficacy of the vaccine and represents the probability of being protected, given that the individual received the vaccine. In the numerical examples, we used two different functions to determine the starting date of administration, namely, Gaussian functions.

**Fig 2:**
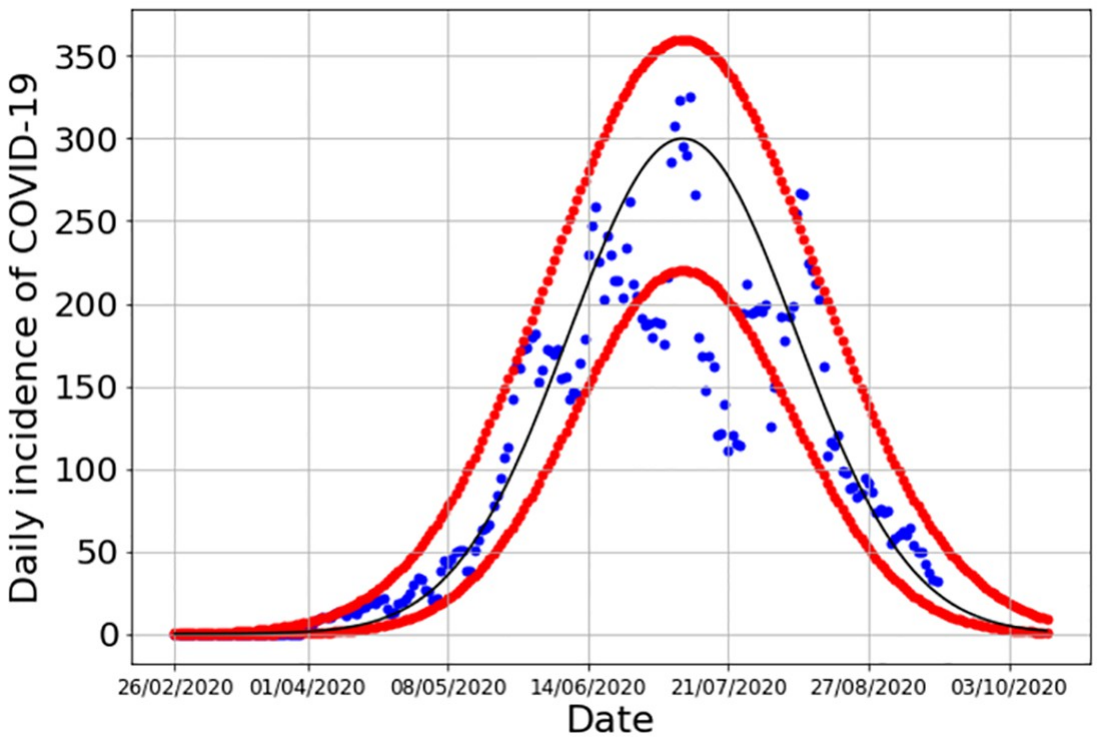
Force of infection in the city of Santos, Southeast Region of Brazil, modeled as a Gaussian function.

The force of infection λ was modeled based in a real case of time-dependent incidence of a COVID-19 outbreak in the city of Santos in the Southeast Region of Brazil in 2020, reproduced in Fig 2.

Considering that the outbreak can be approximated by a Gaussian function, we assume that the force of infection λ is proportional to the following:

$$\begin{array}{c} Incidence\left(t\right)=\chi \frac{1}{\varsigma \sqrt{2\pi }}\text{exp}[\frac{-{\left(t-\tau \right)}^{2}}{2\varsigma^{2}}], \end{array}$$ (15)

where *χ* is the intensity of the outbreak, *τ* is the time delay from the CT launch to the outbreak peak, and *ς* is half of its duration. The values of the parameters in Eq (15) will be given later. Thus, the force of infection of the outbreak λ is defined as the *per capita* incidence, in other words,

$$\begin{array}{c} \lambda \left(t\right)=\frac{Incidence(t)}{S_{\text{Santos}}\left(t\right)}, \end{array}$$ (16)

where *S*_*Santos*_ is the population of the city whose outbreak is shown in Fig 2.

As explained above, the vaccine efficacy, *VE*, is calculated using Eq (1) and the experimental results represented by the quantities in Table 1. Such quantities are translated into the model variables as follows,

$$\begin{array}{c} a=I_{v}\left(t\right),\;b=P\left(t\right)+F\left(t\right),\;c=I_{p}\left(t\right),\;\;\text{and}\;\;d=Pl\left(t\right). \end{array}$$

Therefore, in terms of the model’s variables we have:

$$\begin{array}{c} ARV\left(t\right)=\frac{I_{v}\left(t\right)}{I_{v}\left(t\right)+P\left(t\right)+F\left(t\right)} \end{array}$$ (17)

and

$$\begin{array}{c} ARU\left(t\right)=\frac{I_{p}\left(t\right)}{I_{p}\left(t\right)+Pl\left(t\right)}. \end{array}$$ (18)

Since the incidence can be written using Eqs (15) and (16), its free parameters assume the following values:

$$\begin{array}{c} \;\chi =22,560,\;\tau =30\;\text{days},\;\;\text{and}\;\;\varsigma =135\;\text{days}\;\text{(will}\;\text{vary}\;\text{in}\;\text{the}\;\text{simulations).} \end{array}$$

**Fig 3:**
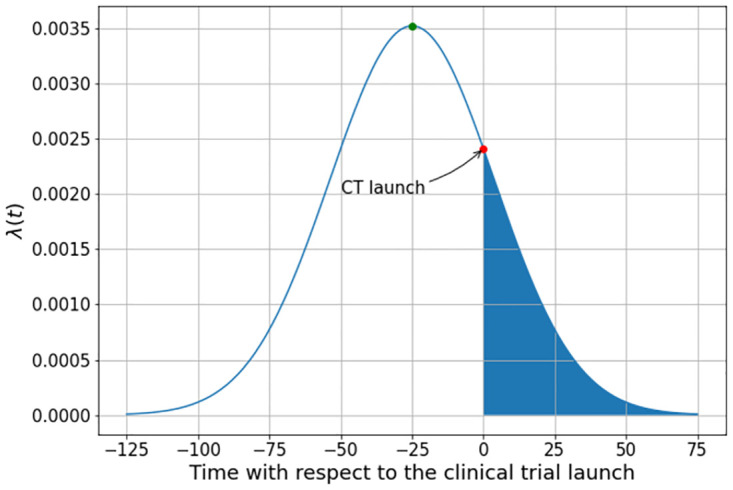
The cohort’s burden of infection during the clinical trial that begins at *t* = 0.

In the following calculations, we shall vary the values of λ(*t*) with respect to the beginning of the trial by changing the position of the peak of the outbreak with respect to the initial time *t* = 0. This procedure is illustrated in Fig 3, where the area below the curve represents the burden of infection *that* the cohort is submitted during the clinical trial at the time *t* = 0. In this figure, the outbreak reaches its peak at time *t* = 25, that is 25 days before the beginning of the trial.

## Results

### The Clinical Trials (CT) ends after the outbreak

We simulated the model in Eqs (7)–(14) for 3 years. We calculate the vaccine efficacy when the trials start at arbitrary times, *t*_*v*_ and *t*_*p*_. We also assume that the position of the outbreak peak varies with respect to the beginning of the trial. The value of VE is calculated at the end of each simulation.

The parameters in these simulated scenarios were set to *q* = 0.5, *ν* = 0.028*days*^−1^, $\chi =0,000352/\sqrt{1650\pi }$, ς=28,7228days, and *τ* assumes different values.

**Fig 4:**
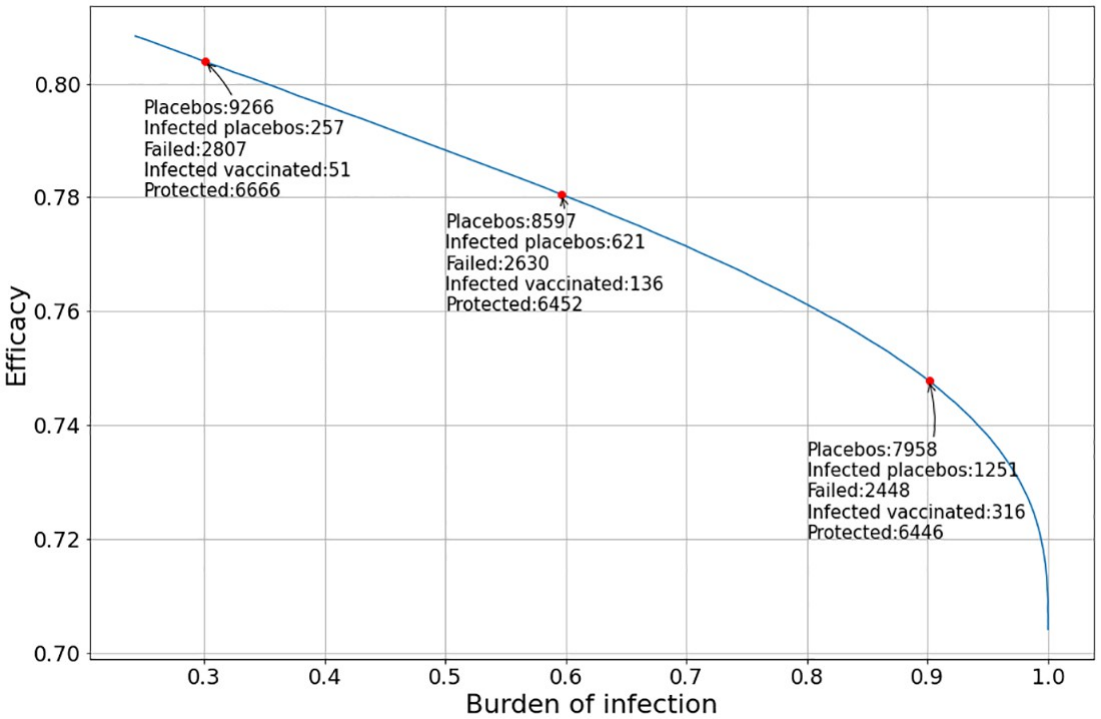
Evolution of the vaccine efficacy as a function of the disease outbreak peak when the vaccine and the placebo inoculation start at *t*_*v*_ = *t*_*p*_ = 0.

Let us consider a set of simulations. In these simulations the position of the peak of the outbreak (given by the value of *τ*) varies with respect to *t* = 0. This means that the burden of incidence to which the trial cohort is subjected may vary from simulation to simulation. In the first simulation we assume that *t*_*v*_ = *t*_*p*_ = 0. The result can be seen in Fig 4.

It can be noted that the value of the vaccine efficacy (VE) varies significantly with the burden of infection suffered by the trial population.

**Fig 5:**
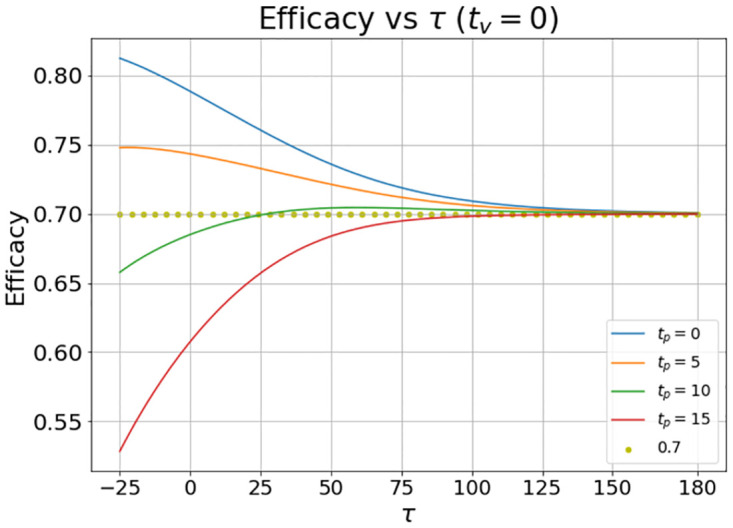
Evolution of the vaccine efficacy as a function of the disease outbreak peak (*τ*) when the vaccine inoculation starts at *t*_*v*_ = 0 and the placebo inoculation starts at *t*_*p*_ = 0, 5, 10, and 15 days. The quantity *τ* is the time delay in days from the clinical trial launch (which is set to *t* = 0) to the outbreak peak.

In Fig 5, which represents the scenario *t*_*v*_ ≤ *t*_*p*_, we show the effect on the calculated VE due to the fact that the placebo administered begins after the vaccine administered, we set *t*_*v*_ = 0 and *t*_*p*_ = 5, 10, and 15 days. The simulation with *t*_*p*_ = 0 is included for comparison.

It is remarkable that the VE is less than the nominal value that was set at *ω* = 0.7 (70%). This is because the value of ARV is reduced by the delay in injecting the placebo.

**Fig 6:**
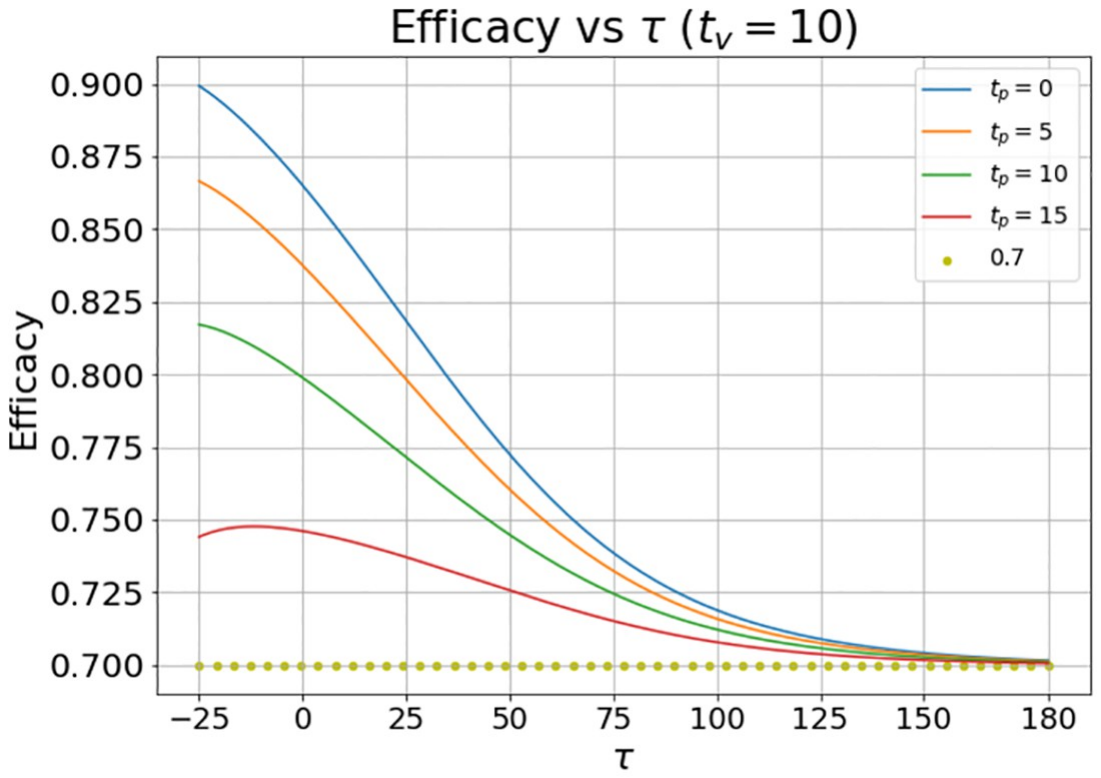
Evolution of the vaccine efficacy as a function of the disease outbreak peak (*τ*) when the vaccine inoculation starts at *t*_*v*_ = 10 and the placebo inoculation starts at *t*_*p*_ = 0, 5, 10, and 15 days. The quantity *τ* is the time delay in days from the clinical trial launch (which is set to *t* = 0) to the outbreak peak.

In Fig 6 the results shown were obtained by setting *t*_*v*_ = 10 days and *t*_*p*_ = 0, 5, 10 and 15 days.

In this case the placebo administered began before the vaccine administered in the first two simulations (with *t*_*p*_ = 0, 5). In this case the calculated VE is generally greater that the nominal value. This is because the value of ARU is reduced by the delay in the administered of the vaccine.

**Fig 7:**
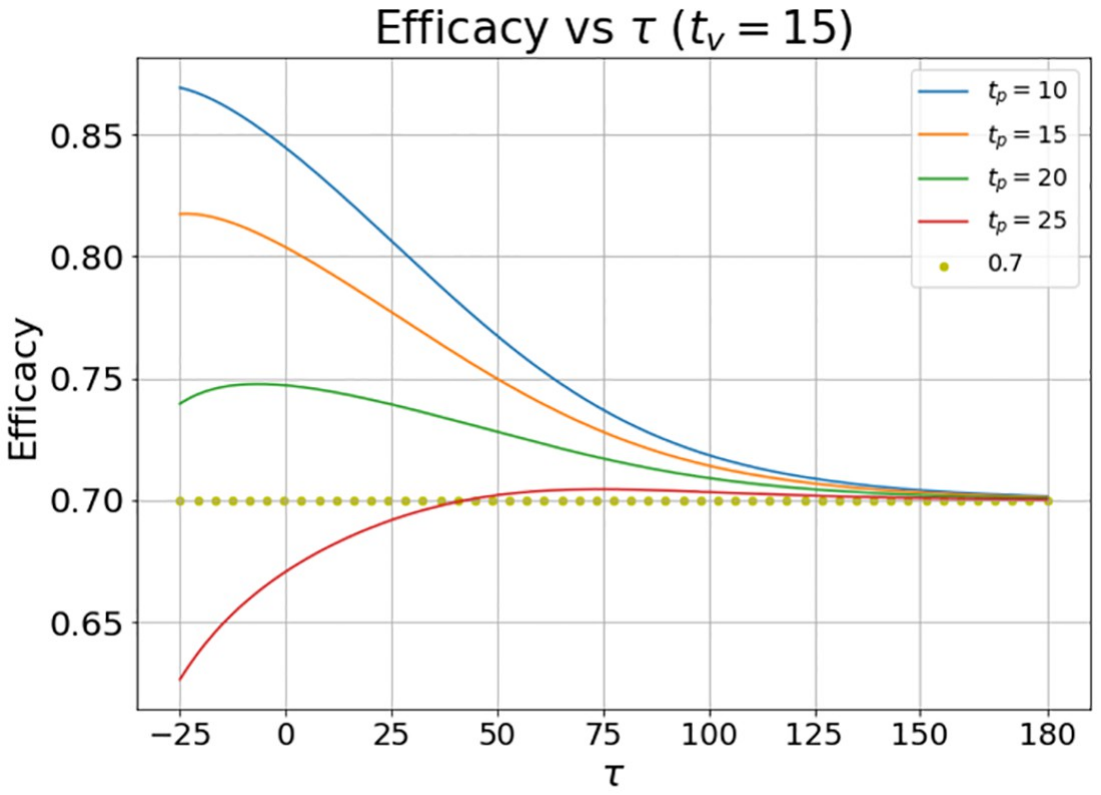
Evolution of the vaccine efficacy as a function of the disease outbreak peak (*τ*) when the vaccine inoculation starts at *t*_*v*_ = 15 and the placebo inoculation starts at *t*_*p*_ = 10, 15, 20, and 25 days. The quantity *τ* is the time delay in days from the clinical trial launch (which is set to *t* = 0) to the outbreak peak.

In Fig 7 the results were obtained when we set *t*_*v*_ = 15 and *t*_*p*_ = 10, 15, 20 and 25 days.

Note that when *t*_*p*_ < *t*_*v*_ the calculated VE is smaller than the nominal value. It is also interesting that when *t*_*p*_ = 20 the calculated VE has a maximum value.

### Clinical trial interrupted when infections reach 200 individuals

In practice, the trial is not as long as assumed in the first set of examples. In the simulations described below the study is designed to be interrupted when the total number of infections attains 200.

**Fig 8:**
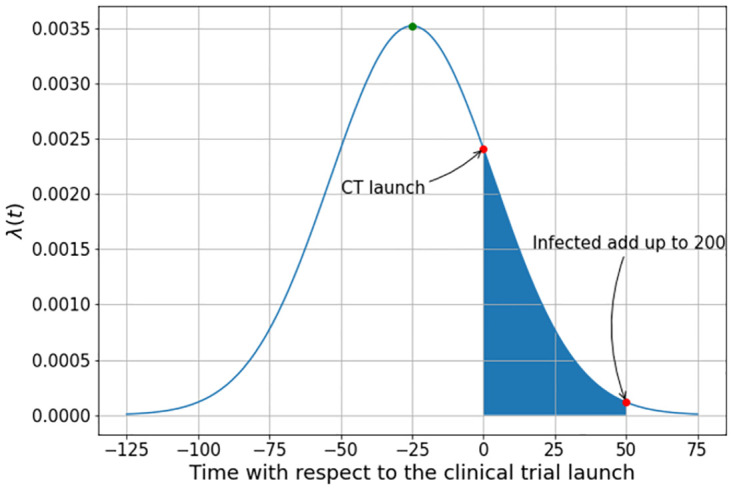
The cohort’s infection burden during the clinical trial.

The idea of interrupting the trial at a certain point is to comply with the common practice in actual clinical trials to obtain the necessary power of test [24]. However, the nominal value of the vaccine efficacy, *ω*, is only reached if at the end of the trial only the compartments *P*, *I*_*v*_ and *I*_*p*_ are different from zero. To do this we run the simulation integrating the incidence curve up to the stopping point show in Fig 8. In other words, the study ends at the first day when the total number of infected individuals inoculated with the vaccine and the placebo reached at least 200.

**Fig 9:**
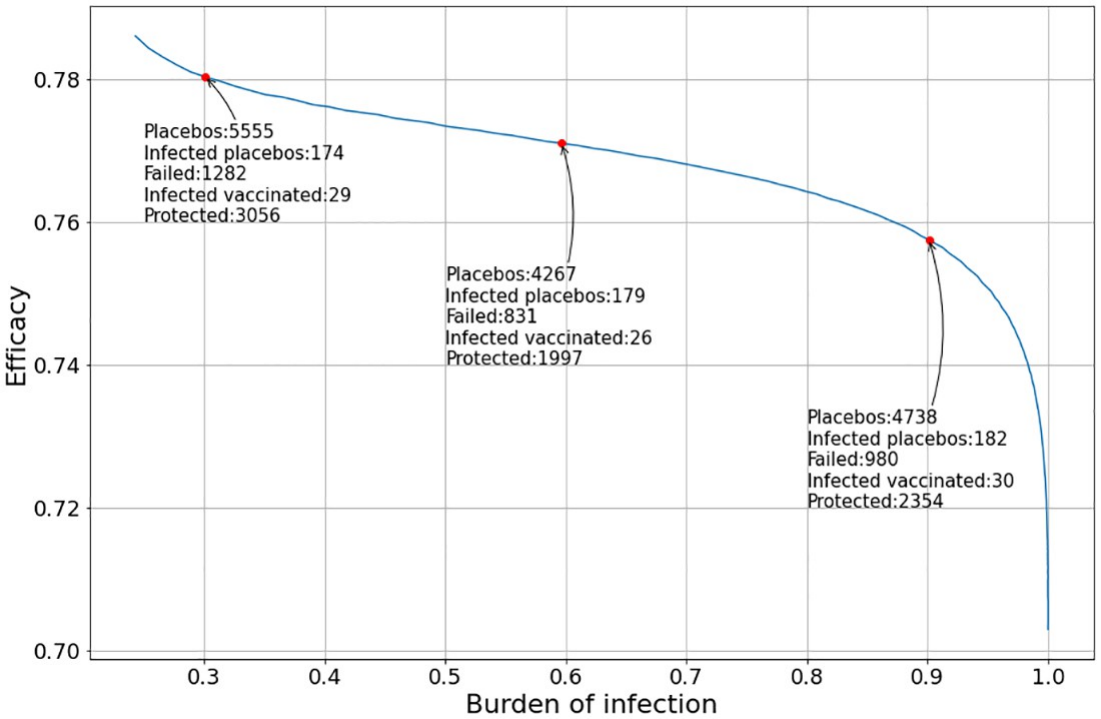
Evolution of the vaccine efficacy as a function of the disease outbreak peak when the vaccine and the placebo inoculation start at *t*_*v*_ = *t*_*p*_ = 0. The clinical trial ends at the first day when the total number of infected individuals inoculated with the vaccine and the placebo reached at least 200.

In Fig 9 we show the evolution of the VE as a functions of the outbreak peak, when *t*_*v*_ = *t*_*p*_ = 0.

Note that the estimated efficacy is different from the nominal efficacy, *w* = 0.7. It is reached when all the compartments of the model but the final ones, are zero. It can be noted that the nominal efficacy is only achieved if the fraction of the burden is sufficient large.

We recall that infected individuals in this study come from three different groups. The ones that were not inoculated (*I*), the inoculated by the placebo (*I*_*p*_) and those inoculated by the vaccine (*I*_*v*_). One natural question is how the non-inoculated perform *vis a vis* those that participated in the study and were inoculated. In particular, what happens to the other compartments when the inoculated individuals reached the value of 200. This is addressed in Table 2 which presents the accumulated numbers of individuals in each compartment of the model in Eqs (7)–(14) after 60 days, 3 years, and at the first day when the total number of infected individuals inoculated with the vaccine and the placebo reached at least 200. In this example, we assume that the vaccine and the placebo inoculation start at *t*_*v*_ = 0, *t*_*p*_ = 0 and the outbreak peak occurs at *τ* = 60.

**Table 2:** Accumulated numbers of individuals in each compartment of the model in Eqs (7)–(13) after 60 days, 3 years, and at the first day when the total number of infected individuals inoculated with the vaccine and the placebo reached at least 200. In this example, the vaccine and the placebo inoculation start at *t*_*v*_ = 0, *t*_*p*_ = 0 and the outbreak peak occurs at *τ* = 60.

Compartment size	60 days	3 years	At the time for which we had at least 200 Inoculated and Infected
Susceptible (*S*)	3299	0	7405
Vaccinated (*V*)	659	0	1340
Placebos (*Pl*)	7201	7798	5890
Infected (*I*)	861	1092	462
Infected placebos (*I*_*p*_)	720	1657	178
Failed (*F*)	2001	2386	1383
Infected vaccinated (*I*_*v*_)	178	451	36
Protected (*P*)	5083	6618	3309

### Vaccination and placebo arms as gaussian distributions

In the first two scenarios we assume that number of injected individuals daily, with the vaccine and the placebo, follows a Gaussian density function, with its mean representing the peak of the trial, and standard deviation representing half of the time duration of the trial. The peak of placebo and vaccine administered does not need to coincide, and using a Gaussian density allows to consider residual administered before and after the trial. Thus, *Θ*_*v*_(*t* − *T*_*v*_) and *Θ*_*p*_(*t* − *T*_*p*_) are replaced by a function *Θ*_*p*_(*s*)*G*(*s*, *L*), where

$$\begin{array}{c} G\left(s,L\right)=\frac{2}{L\sqrt{2\pi }}\text{exp}[-\frac{s^{2}}{2L^{2}}], \end{array}$$ (19)

and *s* = *t* − *T*_*v*_ or *s* = *t* − *T*_*p*_.

We consider three different scenarios:

1. *T*_*v*_ = 0, *T*_*p*_ = 0, and *L* = 20.
2. *T*_*v*_ = 0, *T*_*p*_ = 10, and *L* = 20.
3. *T*_*v*_ = 10, *T*_*p*_ = 0, and *L* = 20.

We evaluate the VE at the first day when the total number of infected individuals inoculated with the vaccine and the placebo reached at least 200.

**Fig 10:**
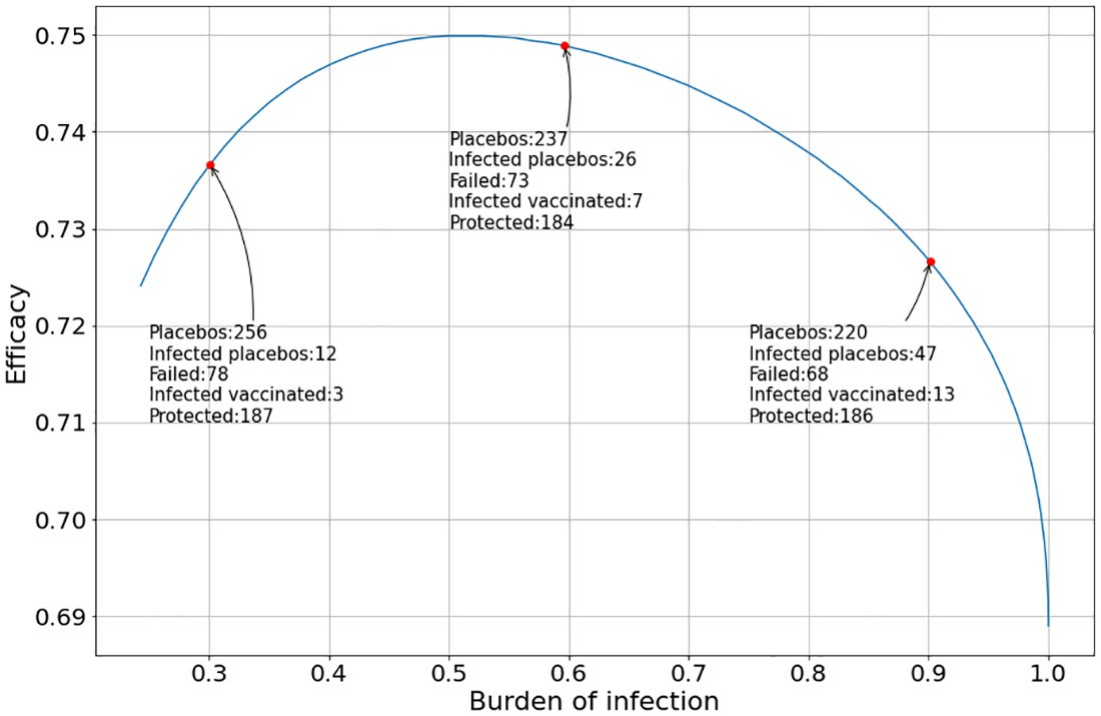
Evolution of the vaccine efficacy as a function of the disease outbreak peak when the peak in the vaccine and the placebo inoculation are at *T*_*v*_ = *T*_*p*_ = 0 and the clinical trial ends at the first day when the total number of infected individuals inoculated with the vaccine and the placebo reached at least 200. The placebo and vaccine daily inoculation follows the Gaussian function in Eq (19).

Fig 10 shows how the VE varies as the outbreak peak changes when the evolution of the Vaccine and the Placebo administered follow the Gaussian density in Eq (19).

**Fig 11:**
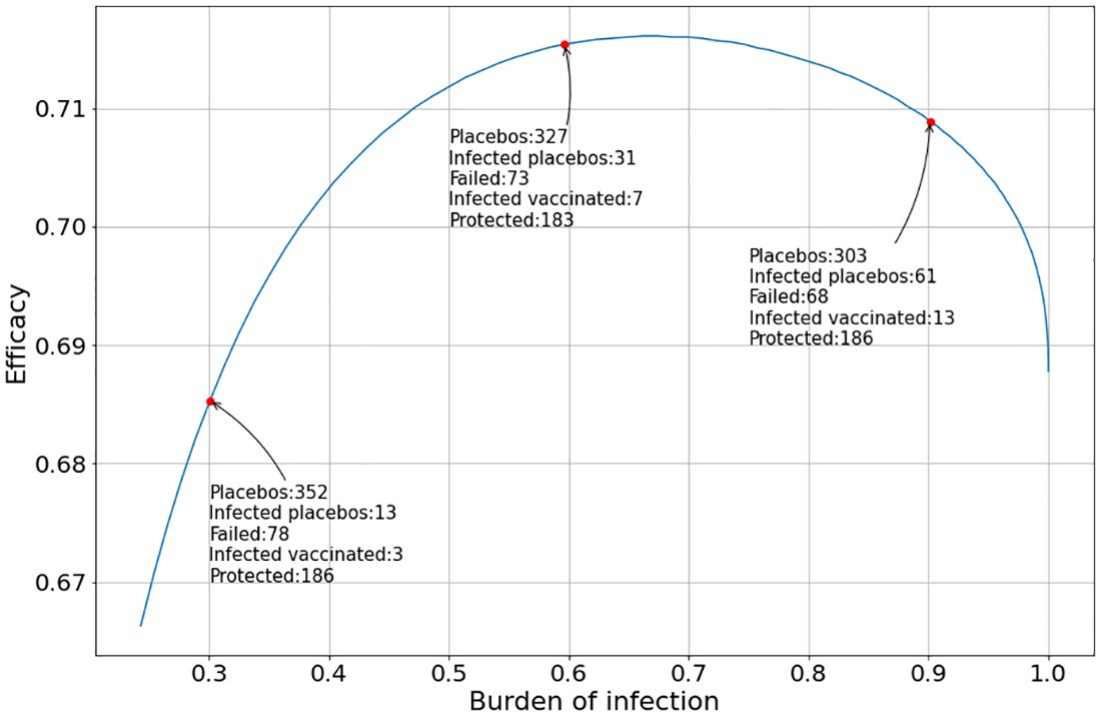
Evolution of the vaccine efficacy as a function of the disease outbreak peak when the peak of the vaccine and the placebo inoculation are at *T*_*v*_ = 0 and *T*_*p*_ = 10, respectively, and the clinical trial ends at the first day when the total number of infected individuals inoculated with the vaccine and the placebo reached at least 200. The placebo and vaccine daily inoculation follows the Gaussian function in Eq (19).

Fig 11 shows the case when the vaccination administered evolution peak is *T*_*v*_ = 0 and the placebo administered peak is *T*_*p*_ = 10.

**Fig 12:**
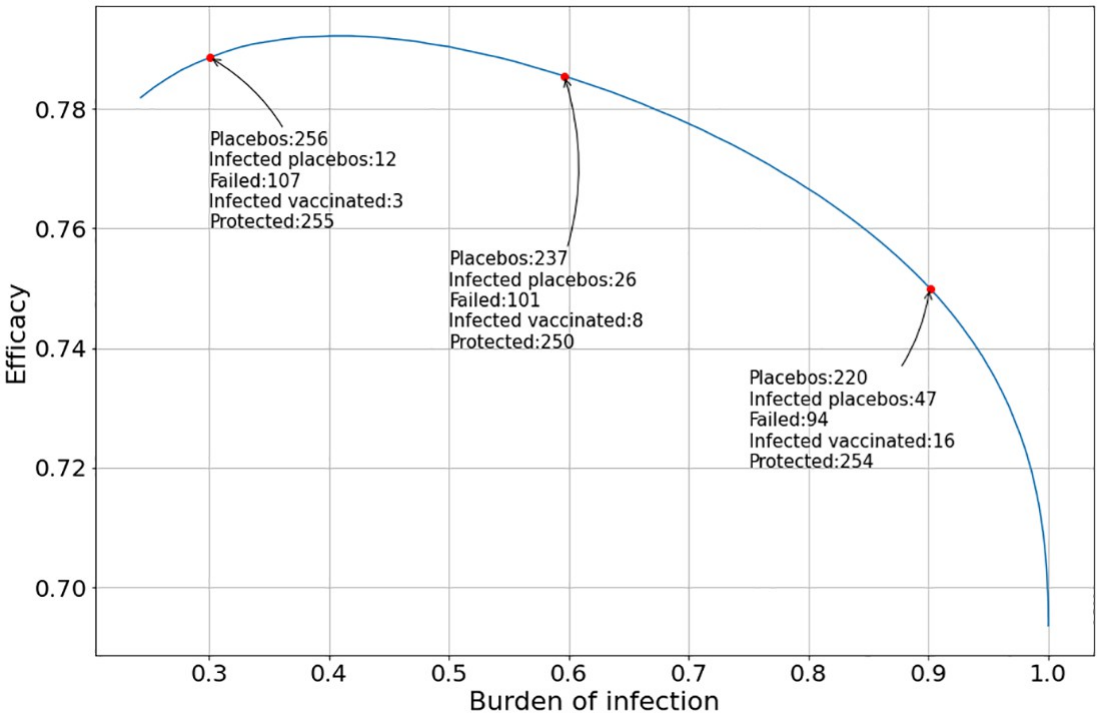
Evolution of the vaccine efficacy as a function of the disease outbreak peak when the peak of the vaccine and the placebo inoculation are at *T*_*v*_ = 10 and *T*_*p*_ = 0, respectively, and the clinical trial ends at the first day when the total number of infected individuals inoculated with the vaccine and the placebo reached at least 200. The placebo and vaccine daily inoculation follows the Gaussian function in Eq (19).

Finally, Fig 12 shows the case when the vaccination administered peak is *T*_*v*_ = 10 and the placebo administered peak is *T*_*p*_ = 0.

### Sensitivity analysis

In what follows, we shall evaluate the sensitivity of an arbitrary dependent variable *Q* with respect to the independent (arbitrary) variable *p*. See [25]. This is done through the calculation of the elasticity defined as

$$\begin{array}{c} \varepsilon_{p}\left[Q\right]=\frac{\partial Q}{\partial p}\frac{p}{Q}\;\text{.} \end{array}$$ (20)

We are going to set *t*_*v*_ = *t*_*p*_ = 0, and since the model is linear, the analytic solution of its states is straightforward. So,

$$\begin{array}{c} S\left(t\right)=S\left(0\right)\text{exp}[-\int_{0}^{t}\left(\lambda \left(s\right)+\nu \right)ds], \end{array}$$ (21)

$$\begin{array}{c} Pl\left(t\right)=S\left(0\right)\left(1-q\right)\text{exp}(-\int_{0}^{t}\lambda \left(s\right)ds)(1-{\text{e}}^{-\nu t}), \end{array}$$ (22)

$$\begin{array}{c} I\left(t\right)=S\left(0\right)\int_{0}^{t}\lambda \left(s\right)\text{exp}[-\int_{0}^{s}\left(\lambda \left(l\right)+\nu \right)dl]ds, \end{array}$$ (23)

$$\begin{array}{c} I_{p}\left(t\right)=S\left(0\right)\left(1-q\right)\int_{0}^{t}\lambda \left(s\right)\text{exp}(-\int_{0}^{s}\lambda \left(l\right)dl)(1-{\text{e}}^{-\nu s})ds, \end{array}$$ (24)

$$\begin{array}{c} V\left(t\right)=S\left(0\right)q\nu {\text{e}}^{-\sigma t}\int_{0}^{t}\text{exp}[-\int_{0}^{s}(\lambda (l)+\nu -\sigma )dl]ds, \end{array}$$ (25)

$$\begin{array}{c} F\left(t\right)=\left(1-w\right)\sigma \text{exp}(-\int_{0}^{t}\lambda \left(s\right)ds)\int_{0}^{t}V\left(s\right)\text{exp}(\int_{0}^{s}\lambda \left(l\right)dl)ds, \end{array}$$ (26)

$$\begin{array}{c} P\left(t\right)=w\sigma \int_{0}^{t}V\left(s\right)ds \end{array}$$ (27)

$$\begin{array}{c} I_{v}\left(t\right)=\int_{0}^{t}\lambda \left(s\right)F\left(s\right)ds. \end{array}$$ (28)

In Table 3, we present the numerical values of the sensitivity *ϵ*_*p*_ using Eqs (21)–(28). For these calculations we take *q* = 0.5, *w* = 0.7, *ν* = 0.028, *σ* = 0.1 and *τ* = 100.

**Table 3:** Sensitivity of the vaccine efficacy with respect to the parameters of the model in Eqs (7)–(14) at different times.

Sensitivity / Time(t)	*t* = 50	*t* = 100	*t* = 150	*t* = 200	*t* = 300	*t* = 500	*t* = ∞
*ε*_*q*_[*VE*]	0.535	0.5092	0.460	0.455	0.4541	0.454	0.454
*ε*_*w*_[*VE*]	1.167	1.209	1.190	1.185	1.1830	1.183	1.183
*ε*_*ν*_[*VE*]	−0.001	−0.019	−0.032	−0.044	−0.050	−0.051	−0.051
*ε*_*σ*_[*VE*]	0.819	1.104	1.163	1.240	1.283	1.288	1.288
*ε*_λ_[*VE*]	−0.583	−0.382	−0.060	−0.001	0	0	0
*ε*_*τ*_[*VE*]	1.626	0.618	0.017	−0.054	−0.054	−0.054	−0.016

More details can be found in the S1 File.

### Illustrating the model with the case of CORONAVAC ^®^ in Brazil

In order to illustrate the model we examine the case of the CT of the vaccine made by firm SINOVAC SARS-CoV-2, called CORONAVAC in Brazil.

The trial was carried out in the second semester of 2020, when there was an outbreak of COVID-19, that begun in March, reaching a peak in May, subsiding from there onward until a second wave begun in late November.

**Fig 13:**
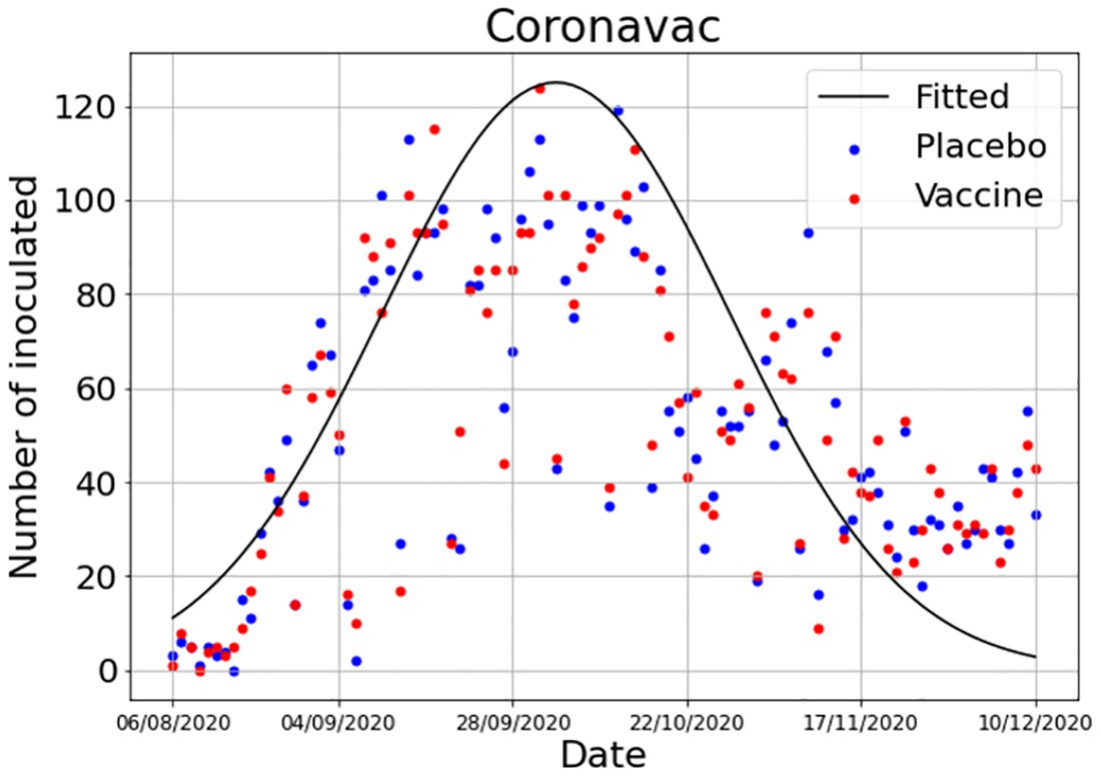
Daily numbers of individuals inoculated with the vaccine and the placebo during the CORONAVAC^®^ trial in Brazil. The solid line represents a continuous function fitted to the data.

We fitted the actual outbreak data with a continuous curve with the same shape as in Eq (15). The result is shown in Fig 13.

The trial began in late July and ended in December. As can be seen from the figure, the trial was conducted in the last quarter of the outbreak. The purpose of the calculations below is to investigate the influence of the period of time elapsed between the peak in the outbreak and the average time the actual trial begun on the estimated efficacy of the vaccine. This period of time was on average equal to 60 days, with variance equal to 30 days. We then simulated the model, varying the value of the proportion *w* (the nominal efficacy) until the calculated efficacy reached the actual value of efficacy founded in the trial, that is, 50.3% against infection. The efficacy obtained should the trial begun at the peak of the outbreak (that is, 60 days before, on average) was 58.8%. This result does not depend on whether the trial lasts 3 years or if it is terminated when total infections reach 200, as was the case of the CORONAVAC trail.

This illustrate in practice the time-dependence of the calculated vaccine efficacy in settings where the force of infection varies with time (an outbreak). Therefore, we must consider this time-dependence when estimating vaccine efficacy in CTs of vaccines against infections that show a substantial time variation or seasonality.

## Discussion

In this paper we tested the hypothesis that the time difference between the moment the CT begins and the peak in the outbreak intensity results in substantially different values for VE. We tested this hypothesis with a mathematical model and exemplify the method with the case of the VE efficacy estimation for one of the vaccines against the new coronavirus SARS-CoV-2. The time-dependence of VE on the force of infection has been previously explored by Kaslov [15] who demonstrated that when the force of infection varies with time, this affects the estimation of VE. This author proposed three different shapes for VE depending on whether the force of infection is constant, or varies linearly with time or varies as a logarithmic fashion with time.

The advent of SARS-CoV-2, however, brought new challenges for the estimation of VE [5, 23], the most important of which, is to estimate VE in the middle of an outbreak. We have shown that, depending on the time difference the peak of the outbreak and the time the CT begins VE can result in substantially different values. Furthermore, this time-dependence of the VE estimation is not linear but assume distinct shapes depending on several factors, in particular if the trial begins before or after the peak of the outbreak. Moreover, VE has shown to be strongly dependent on the duration of the trial. As shown in the sensitivity analysis, VE shown a variable sensitivity to each of the parameters that remarkably varies with the duration of the trial. This explains the strange shapes of the figures that relate VE with the burden of infection. As we can note in Table 3, VE is much more sensitive to the period between the peak of the outbreak and the beginning of the trial, expressed in the value of the parameter *τ*. Sensitivity of VE is 5 times more influenced by *τ* when the trial lasts only 50 days than when the trial lasts 500 days. Moreover, the relative sensitivity of VE to each of the parameters also varies with the duration of the trial. For instance, VE is much more sensitive to the rate of vaccination *ν* than to the nominal efficacy *w* when the trial lasts 50 days but it reverses when the trial lasts more than 100 days. In addition, it is noteworthy that VE is not sensitive at all to the force of infection λ when the trial lasts more than 300 days. However, one should be aware that this sensitivity is related to the magnitude of the force of infection, not to its time-dependence.

Some important limitations of the model should me mentioned here. First, choosing the definition of vaccine efficacy based on risk rather than rate may not be a good option. The risk ratio, as it aggregates over time, cannot discriminate different temporal trajectories that lead to the same final risk. that is, there is an infinite number of possibilities to obtain the same value of vaccine efficacy, when measured from the risk ratio, which could be discriminated if they were measured as a ratio of rates or comparison of survival curves [26].

In fact, the vast majority of vaccine efficacy studies use Poisson regression or survival analysis as statistical models. These approaches make it possible to express efficiency from the ratio of rates, which can be a time-dependent quantity. However, these techniques are more appropriate to estimate post-license efficiency rather than efficacy as is the case of our model. The two approaches above (rate and risk) may still be unsatisfactory because they do not allow the logically correct comparison to be made. Ideally, the comparison that makes sense to is between an individual vaccinated and challenged with a standard inoculum and an individual who has taken the placebo and is challenged with the same inoculum. The above measures do not differentiate the number of challenges vaccinated and unvaccinated have been exposed to. some of these individuals, the majority, do not receive any inoculum and are therefore uninformative. other individuals receive 2 or more inocula and this will complicate the interpretation of the vaccine efficacies defined above [27]. Finally, it should be mentioned that we did not take into account the fact pointed by Hay et al. [28], who showed that during the growing phase of the outbreak there could be a higher proportion of individuals with higher viral loads, whereas during the declining phase of the outbreak there would be more individuals with lower viral loads. This would probably influence our results and in a future work we will address this issue.

## Supporting information

S1 File This file contains the sensitivity analysis of VE with respect to the model parameters, a list of symbols, and the VE accounting for a time-dependent force of infection. (PDF)
