# Supplementary material for: Time-dependent vaccine efficacy estimation quantified by a mathematical model
Source: PLoS One. 2023 May 11;18(5):e0285466. doi: 10.1371/journal.pone.0285466 (PMC10174497; doi:10.1371/journal.pone.0285466)
Supplement: S1 File — (PDF) [file pone.0285466.s001.pdf]

Supplementary Materials to: Time-Dependent Vaccine Efficacy Estimation Quantified by a Mathematical Model

Jennifer Loria[1,2], Vinicius Albani[3], Francisco A. B. Coutinho[4], Dimas T. Covas,[5], Claudio J. Struchiner[6], Jorge P. Zubelli[7,*], and Eduardo Massad[6,8]

[1]Instituto de Matemática Pura e Aplicada, Rio de Janeiro, Brazil
[2]School of Mathematics, Universidad de Costa Rica, San José, Costa Rica
[3]LAMMCA, Department of Mathematics, Federal University of Santa Catarina, Florianopolis, Brazil
[4]Department of Pathology, University of São Paulo, Brazil
[5]Instituto Butantan, São Paulo, Brazil
[6]School of Applied Mathematics, Fundação Getúlio Vargas, Rio de Janeiro, Brazil
[7]Mathematics Department, Khalifa University, Abu Dhabi, UAE
[8]School of Medicine, University of São Paulo and LIM01-HCFMUSP, São Paulo, Brazil
[*]Corresponding author,jorge.zubelli@ku.ac.ae

S.1 Sensitivity Analysis

We present the computation of the sensitivity of VE w.r.t. the parameters $q, w, \nu, \sigma, \lambda$ and τ.

$$\frac{\partial(VE)}{\partial\rho} = \frac{I_v P_l(I_p'(I_v+F)+I_pP')}{I_p^2(I_v+F+P)^2} + \frac{(I_p+P_l)(I_v(P'+F')-I_v'(P+F))}{I_p(I_v+F+P)^2} - \frac{I_vP_l}{I_p(I_v+F+P)}$$

Here, ρ is any of the independent variables $q, w, \nu, \sigma, \lambda$ or τ. Using Eq. (20), we get

$$\varepsilon_\rho(VE) = \left(\frac{I_vP_l(I_p'(I_v+F)+I_pP')}{I_p(I_v+F+P)} + \frac{(I_p+P_l)(I_v(P'+F')-I_v'(P+F))}{(I_v+F+P)} - I_vP_l'\right)\frac{\rho}{I_p(P+F)-I_vP_l}$$

Now, to determine the sensitivity of each parameter in vacinal efficacy, we must take into account that for each of these parameters we must determine at least 5 derivatives of the 5 compartments involved in the function $VE(t)$, plus the derivative of $V(t)$ since it interferes with the other compartments.

We will present the final results of the derivatives:

$\rho = q$

$$\frac{\partial Pl}{\partial q} = -S(0)\exp\left(-\int_0^t \lambda(s)ds\right)\left(1-\mathrm{e}^{-\nu t}\right),$$
$$\frac{\partial I_p}{\partial q} = -S(0)\int_0^t \lambda(s)\exp\left(-\int_0^s \lambda(l)dl\right)\left(1-\mathrm{e}^{-\nu s}\right)ds,$$
$$\frac{\partial V}{\partial q} = S(0)\nu\mathrm{e}^{-\sigma t}\int_0^t \exp\left[-\int_0^s \left(\lambda(l)+\nu-\sigma\right)dl\right]ds,$$
$$\frac{\partial F}{\partial q} = (1-w)\sigma\exp\left(-\int_0^t \lambda(s)ds\right)\int_0^t \frac{\partial V}{\partial q}(s)\exp\left(\int_0^s \lambda(l)dl\right)ds,$$
$$\frac{\partial P}{\partial q} = w\sigma\int_0^t \frac{\partial V}{\partial q}(s)ds$$
$$\frac{\partial I_v}{\partial q} = \int_0^t \lambda(s)\frac{\partial F}{\partial q}(s)ds.$$

$\rho = w$

$$\frac{\partial Pl}{\partial w} = 0$$
$$\frac{\partial I_p}{\partial w} = 0$$
$$\frac{\partial V}{\partial w} = 0$$
$$\frac{\partial F}{\partial w} = -\sigma\exp\left(-\int_0^t \lambda(s)ds\right)\int_0^t V(s)\exp\left(\int_0^s \lambda(l)dl\right)ds,$$
$$\frac{\partial P}{\partial w} = \sigma\int_0^t V(s)ds$$
$$\frac{\partial I_v}{\partial w} = \int_0^t \lambda(s)\frac{\partial F}{\partial w}(s)ds.$$

$\rho = \nu$

$$\frac{\partial Pl}{\partial \nu} = t(1-q)S(t),$$
$$\frac{\partial I_p}{\partial \nu} = \int_0^t \lambda(s)\frac{\partial Pl}{\partial \nu}(s)ds,$$

$$\frac{\partial V}{\partial \nu} = S(0)q\mathrm{e}^{-\sigma t}\left(\int_0^t \exp\left[-\int_0^s \left(\lambda(l)+\nu-\sigma\right)dl\right]ds - \nu\int_0^t s\exp\left[-\int_0^s \left(\lambda(l)+\nu-\sigma\right)dl\right]ds\right),$$

$$\frac{\partial F}{\partial \nu} = (1-w)\sigma\exp\left(-\int_0^t \lambda(s)ds\right)\int_0^t \frac{\partial V}{\partial \nu}(s)\exp\left(\int_0^s \lambda(l)dl\right)ds,$$

$$\frac{\partial P}{\partial \nu} = w\sigma\int_0^t \frac{\partial V}{\partial \nu}(s)ds$$

$$\frac{\partial I_v}{\partial \nu} = \int_0^t \lambda(s)\frac{\partial F}{\partial \nu}(s)ds.$$

$\rho = \sigma$

$$\frac{\partial Pl}{\partial \sigma} = 0,$$

$$\frac{\partial I_p}{\partial \sigma} = 0,$$

$$\frac{\partial V}{\partial \sigma} = S(0)q\nu\mathrm{e}^{-\sigma t}\left(\int_0^t s\exp\left[-\int_0^s \left(\lambda(l)+\nu-\sigma\right)dl\right]ds - \sigma\int_0^t \exp\left[-\int_0^s \left(\lambda(l)+\nu-\sigma\right)dl\right]ds\right),$$

$$\frac{\partial F}{\partial \sigma} = (1-w)\exp\left(-\int_0^t \lambda(s)ds\right)\left(\int_0^t V(s)\exp\left(\int_0^s \lambda(l)dl\right)ds + \sigma\int_0^t \frac{\partial V}{\partial \sigma}(s)\exp\left(\int_0^s \lambda(l)dl\right)ds\right),$$

$$\frac{\partial P}{\partial \sigma} = w\int_0^t V(s)ds + w\sigma\int_0^t \frac{\partial V}{\partial \sigma}(s)ds$$

$$\frac{\partial I_v}{\partial \sigma} = \int_0^t \lambda(s)\frac{\partial F}{\partial \sigma}(s)ds.$$

$\rho = \lambda$

In this case, we treat λ as if it were a constant when performing the derivation:

$$
\begin{aligned}
\frac{\partial P_l}{\partial \lambda} &= -tP_l(t), \\
\frac{\partial I_p}{\partial \lambda} &= \int_0^t \left(P_l(s) + s\lambda(s)P_l(s)\right) ds, \\
\frac{\partial V}{\partial \lambda} &= -S(0)q\nu \mathrm{e}^{-\sigma t} \int_0^t s \exp\left[-\int_0^s \left(\lambda(l) + \nu - \sigma\right) dl\right] ds, \\
\frac{\partial F}{\partial \lambda} &= -tF(t) + (1-w)\sigma \exp\left(-\int_0^t \lambda(s) ds\right) \int_0^t \exp\left(\int_0^s \lambda(l) dl\right) \left[\frac{\partial V}{\partial \lambda}(s) + sV(s)\right] ds, \\
\frac{\partial P}{\partial \lambda} &= w\sigma \int_0^t \frac{\partial V}{\partial \lambda}(s) ds \\
\frac{\partial I_v}{\partial \lambda} &= \int_0^t \left[\lambda(s)\frac{\partial F}{\partial \lambda}(s) + F(s)\right] ds.
\end{aligned}
$$

$\rho = \tau$

In this part we need one more derivative than in the previous cases because the function λ has τ as one of its parameters.

$$\frac{\partial \lambda}{\partial \tau} = \lambda(t)\frac{(t-\tau)}{\varsigma^2},$$
$$\frac{\partial P_l}{\partial \tau} = -P_l(t)\int_0^t \frac{\partial \lambda}{\partial \tau}(s)ds,$$
$$\frac{\partial I_p}{\partial \tau} = \int_0^t \left[\frac{\partial \lambda}{\partial \tau}(s)P_l(s) + \lambda(s)\frac{\partial P_l}{\partial \tau}(s)\right] ds,$$
$$\frac{\partial V}{\partial \tau} = -S(0)q\nu \mathrm{e}^{-\sigma t}\int_0^t \exp\left[-\int_0^s (\lambda(l)+\nu-\sigma)\, dl\right]\left(\int_0^s \frac{\partial \lambda}{\partial \tau}(l)dl\right) ds,$$
$$\frac{\partial F}{\partial \tau} = -F(t)\int_0^t \frac{\partial \lambda}{\partial \tau}(s)ds +$$
$$(1-w)\sigma \exp\left(-\int_0^t \lambda(s)ds\right)\int_0^t \exp\left(\int_0^s \lambda(l)dl\right)\left[\frac{\partial V}{\partial \tau}(s) + V(s)\left(\int_0^s \frac{\partial \lambda}{\partial \tau}(l)dl\right)\right] ds,$$
$$\frac{\partial P}{\partial \tau} = w\sigma \int_0^t \frac{\partial V}{\partial \tau}(s)ds$$
$$\frac{\partial I_v}{\partial \tau} = \int_0^t \left[\lambda(s)\frac{\partial F}{\partial \tau}(s) + F(s)\frac{\partial \lambda}{\partial \tau}(s)\right] ds.$$

S.2 Nomenclature and List of Symbols

CT	Clinical trial.
VE	Vaccine efficacy.
COVID-19	Coronavirus Disease 2019.
RR	Relative risk.
ARV	Infected fraction among vaccinated.
ARU	Infected fraction among unvaccinated.
SARS-CoV-2	Severe acute respiratory syndrome Coronavirus 2.
$S(0)$	Number of individuals in the volunteer group who never had the disease.
q	Fraction of individuals that will be injected with the vaccine.
τ	The time delay from the clinical trial launch to the outbreak peak.
a	Number of individuals inoculated with the vaccine and infected.
b	Number of individuals inoculated with the vaccine and not infected.
c	Number of individuals inoculated with the placebo and infected.
d	Number of individuals inoculated with the placebo and not infected.
N_v	Total number of individuals inoculated with the vaccine.
N_p	Total number of individuals inoculated with the placebo.
λ_v	The force of infection among vaccinated individuals.
λ_p	The force of infection among those inoculated with the placebo.
S	Susceptible individual.
Pl	Individuals inoculated with the placebo.
I_p	Infected individuals inoculated with the placebo.
I	Infected individuals who did not receive the vaccine nor the placebo.
V	Individuals inoculated with the vaccine.
P	Vaccinated individuals that acquired immunity against the disease.
F	Vaccinated individuals that did not acquire immunity against the disease.
I_v	Infected individuals inoculated with the vaccine.
w	Ground truth vaccine efficacy.
$1/\sigma$	Average time of immunization after vaccination.
ν	Daily rate of inoculation.
λ	The force of infection in the mathematical model.
$\Theta_v(s)$	Determines the vaccination period.

$\Theta_p(s)$	Determines the placebo inoculation period.
t_v	The time when the vaccine starts to be injected.
T_v	The time when the vaccine inoculation attains its peak.
t_p	The time when the placebo starts to be injected.
T_p	The time when the placebo inoculation attains its peak.
ε	Sensitivity of the vaccine efficacy.

S.3 Time-Dependent Force of Infection

If λ_v and λ_p are time-dependent, the argument is the same with λ_p and λ_p replaced by $\int_0^t \lambda_v(s)ds$ and $\int_0^t \lambda_p(s)ds$ respectively.

Since becoming infected is a random event, both ARV and ARU correspond to probabilities. In fact, both can be modelled as Poisson stochastic processes. So let ARV be the following probability:

$$\begin{aligned} ARV = & \text{ Probability of } n \text{ infected among vaccinated} \\ & = p_n(t) = \mathrm{e}^{-\int_0^t \lambda_v(s)ds} \frac{\left(\int_0^t \lambda_v(s)ds\right)^n}{n!}, \end{aligned} \quad \text{(S.1)}$$

and ARU has an analogous formula

$$\begin{aligned} ARU = & \text{ Probability of } m \text{ infected among unvaccinated} \\ & = p_m(t) = \mathrm{e}^{-\int_0^t \lambda_p(s)ds} \frac{\left(\int_0^t \lambda_p(s)ds\right)^m}{m!}. \end{aligned} \quad \text{(S.2)}$$

Now, we must calculate the probability that VE, defined by Eq. (1), is between 0 and 1. Or alternatively, we must evaluate the probability that $Z = p_n(t)/p_m(t)$ is between 0 and 1.

We use a method proposed in Amaku et al. [1],

$$\begin{aligned} Probability_{\frac{ARV}{ARU}}\left(Z = \frac{n}{m}\right) &= P_{\frac{ARV}{ARU}}(Z) \\ &= \mathrm{e}^{-\int_0^t (\lambda_p(s)+\lambda_v(s))ds} \sum_{m=1}^{M} \frac{\left(\int_0^t \lambda_p(s)ds\right)^m \left(\int_0^t \lambda_v(s)ds\right)^{Zm}}{m!(Zm)!}, \end{aligned} \quad \text{(S.3)}$$

where n and m are integer numbers and Z is rational. The number M is N_v or N_p, whichever is smaller.

References

[1] Amaku M, Coutinho FAB, Raimundo SM, Lopez LF, Nascimento Burattini M, Massad E. A comparative analysis of the relative efficacy of vector-control strategies against dengue fever. Bulletin of Mathematical Biology. 2014;76(3):697–717.
